# Necrotizing Fasciitis Secondary to Mycotic Femoral Aneurysm: A Narrative Review of Diagnosis and Management Strategies

**DOI:** 10.7759/cureus.37586

**Published:** 2023-04-14

**Authors:** David Elkhoury, Sarah Quick, Amy E Kalloo, Vasavi Rakesh Gorantla

**Affiliations:** 1 Anatomical Sciences, St. George's University School of Medicine, St. George, GRD; 2 Surgery, St. George's University School of Medicine, St. George, GRD; 3 Clinical Sciences, St. George's University, St. George, GRD

**Keywords:** polymicrobial infection, iv drug abusers, infective endocarditis, femoral aneurysm, mycotic, necrotizing fasciitis

## Abstract

This comprehensive literature review aims to investigate the pathophysiology, clinical manifestations, diagnostic tools, and treatment options for necrotizing fasciitis secondary to mycotic femoral aneurysm, a rare and potentially lethal infectious disease, particularly focusing on any changes throughout the years for an update of the current literature. The pathophysiology of necrotizing fasciitis and mycotic femoral aneurysms is a complex and multifaceted process that typically involves bacterial infections as a common precursor to the onset of these conditions. This can potentially lead to the formation of an aneurysm. As the infection progresses, it can spread from the aneurysm to surrounding soft tissues, resulting in significant tissue damage, obstructed blood circulation, and ultimately culminating in cell death and necrosis. Clinical manifestations of these conditions are diverse and encompass a range of symptoms, such as fever, localized pain, inflammation, skin changes, and other indicators. It is worth noting that skin color can influence the presentation of these conditions, and in patients with diverse skin tones, certain symptoms may be less noticeable due to a lack of visible discoloration. Imaging, laboratory findings, and clinical presentation are important components of the diagnosis of mycotic aneurysms. CT scans are a reliable tool for identifying specific features of infected femoral aneurysms, and elevated inflammatory laboratory results can also suggest a mycotic aneurysm. In the case of necrotizing fasciitis, clinicians should maintain a high level of suspicion as this condition is rare but life-threatening. Clinicians will need to view the big picture when an infection may be caused by necrotizing fasciitis, considering CT imaging, blood work, and clinical presentation of the patient without delaying surgical intervention. By incorporating the diagnostic tools and treatment options outlined in this review, healthcare professionals can improve patient outcomes and reduce the burden of this rare and potentially lethal infectious disease.

## Introduction and background

Introduction

Necrotizing fasciitis is the most common necrotizing soft tissue infection, defined as a rapidly progressing infection typically resulting in necrosis of superficial and deep fascia as well as surrounding tissue [1]. In this paper, we look at necrotizing fasciitis secondary to mycotic femoral aneurysms, its pathogenesis, and pathophysiology along with current management techniques. While its name indicates that it is mycotic in nature, it is indeed a misnomer. Mycotic in this case simply describes the morphological structure of the aneurysm itself, because of its mushroom shape. These types of infected vessels can be bacterial, viral, or fungal in origin with Streptococcus pyogenes and Staphylococcus aureus being the most commonly implicated pathogens [2].

Contrast CT is the most effective imaging modality for detecting aneurysms. The treatment of mycotic aneurysms involves surgical intervention followed by antibiotic treatment [2]. Necrotizing fasciitis is as common as 1 in every 100,000 people worldwide, however, in the United States, this number drops to 0.4 instead of 1 [1]. To go further, necrotizing fasciitis secondary to a mycotic femoral aneurysm is an even rarer disease with only 3% of aneurysms becoming infected [3]. Risk factors for necrotizing fasciitis secondary to mycotic femoral aneurysm include infective endocarditis most commonly with intravenous drug abusers (IVDA), surgery, especially stenting procedures and transplants, and other nonspecific diseases such as diabetes mellitus, immunosuppression, liver cirrhosis, end-stage renal disease, pulmonary diseases, and malignancies, with isolated femoral aneurysms commonly occurring in men compared to women attributed to an increased incidence of atherosclerosis in the male sex. Other causes specific to isolated femoral aneurysm formation includes IV drug use, smoking, and percutaneous arterial access [2,4]. The pathophysiology of this disease is complex and multifactorial but ultimately comes down to several different cellular and molecular processes with bacterial infection being the most common precipitator [5,6]. A definitive diagnosis of necrotizing fasciitis is exploratory surgery; however, imaging and lab work can be used to differentiate necrotizing fasciitis from other infections [7]. The management of necrotizing fasciitis can be tailored to each individual case and can involve surgical debridement, aggressive antibiotic therapy, and maximal oxygenation of tissue, with or without amputation [2,8].

Methods

A comprehensive literature search was conducted using various electronic databases including PubMed, ScienceDirect, and Google Scholar to retrieve reviews, experimental studies, and meta-analyses. The search of databases was limited to articles written between 1993 and 2023, utilizing the search terms “necrotizing fasciitis,” “femoral artery” and “aneurysm.” Search outcomes were compared using a PICO (Population, Intervention, Comparison, and Outcome) framework. Exclusion criteria included articles written in languages other than English. Ultimately the authors selected 32 articles for inclusion based on relevance, findings, and source quality.

Bias

The main advantage of this research process includes the thorough, independent reviews conducted by each author of the literature that was selected. This process attempted to minimize as much reviewer and selection bias as possible.

Disadvantages remain, however, inclusive of ruling out bias completely due to the limited number of research articles that exist as a result of low disease incidence. As with most review papers, this one is deficient in originality when compared with experimental studies as no hypothesis testing was involved.

Pathophysiology of Mycotic Femoral Aneurysm and Necrotizing Fasciitis

The pathophysiology of mycotic femoral aneurysms and necrotizing fasciitis is complex and multifactorial, involving a range of cellular and molecular processes. Although the exact etiology of necrotizing fasciitis remains unclear, it is generally accepted that bacterial infection is a common precipitating factor [5,6].

According to current medical literature, it is widely posited that a mycotic femoral aneurysm has the potential to elicit detrimental effects on the intimal layer, which is the innermost lining of the artery comprised of endothelial cells. Intima infection is the outcome of both bacterial seeding and arterial damage. Bacterial seeding occurs when bacteria enter the bloodstream and travel to the site of the femoral artery, where they can adhere to the damaged intima layer and begin to multiply. As the microorganisms penetrate deeper into the vessel wall, they can rapidly break down the underlying layers, leading to the development of an aneurysm [9]. In response to bacterial infections, pro-inflammatory cytokines are released, and attract neutrophils. Together, they induce the vessel wall to break down by activating matrix metalloproteinases, enzymes capable of breaking down the extracellular matrix of the vessel wall [10].

**Figure 1:**
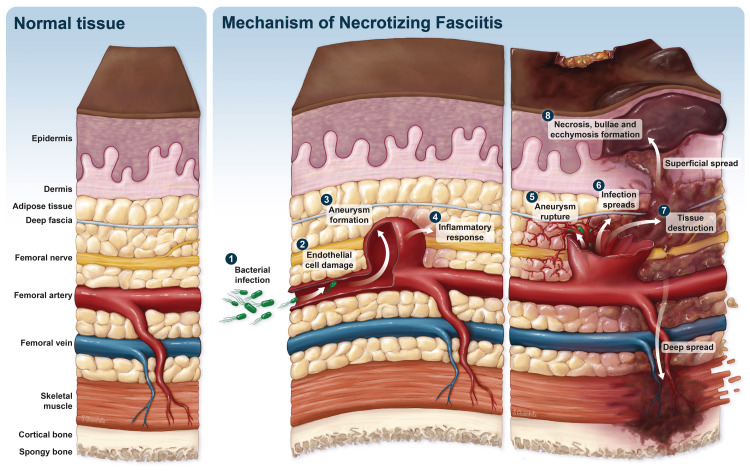
Normal Tissue and Mechanism of Necrotizing Fasciitis Secondary to Mycotic Femoral Aneurysm. Figure 1 depicts normal tissue and mechanism of necrotizing fasciitis secondary to mycotic femoral aneurysm [12]. Reproduced after seeking permission from Massachusetts Medical Society (receipt available upon request).

The spread of infection from the aneurysm into the surrounding soft tissues can result in the destruction of tissue structures, leading to a range of clinical manifestations, as illustrated in Figure 1. Bacteria that proliferate within the superficial fascia can release a combination of enzymes, endotoxins, and exotoxins that can contribute to the progression of infection into the fascia [11]. The consequences of this progression can lead to reduced microcirculation, tissue ischemia, and, ultimately, cell death and necrosis in the affected tissues. The fascial plane, which has a limited blood supply, often serves as the conduit for the spread of infection. The superficial tissues may initially appear unaffected, which can delay the diagnosis and surgical treatment of the condition [1]. This underscores the importance of early detection and prompt management. Mechanism of necrotizing fasciitis secondary to mycotic femoral aneurysm is depicted in Figure 1.

Clinical Manifestations of Mycotic Femoral Aneurysm and Necrotizing Fasciitis

The most frequent manifestations of an infected aneurysm are typically characterized by several common symptoms, such as fever, localized pain, inflammation in the affected artery, and the presence of a pulsatile mass [13]. However, because these symptoms are not specific to an infected aneurysm and lack of specificity, these symptoms might result in the incorrect diagnosis of an unidentified fever. Consequently, patients may remain undiagnosed until they experience severe symptoms, including sepsis, hemorrhage, thrombosis, or rupture. According to Patra et al. [14], 83% of patients with infected aneurysms experience both discomfort and fever. Pain often results from the rapid expansion of the aneurysm and may be accompanied by local inflammation during physical examination, such as the presence of a bruit, which occurs in approximately 50% of cases [15].

In cases of necrotizing fasciitis, tenderness upon palpation extending beyond the erythematous border, cellulitis, and crepitus may be observed during physical examination. Additional potential indicators of necrotizing fasciitis may include ecchymotic skin changes, bullae, paresthesia, and dysesthesia. Crepitus and subcutaneous emphysema are commonly present [16].

In the clinical setting, the recognition of skin color as a key factor in evaluating patients, particularly those with diverse skin tones, is crucial. Skin color has a significant impact on the presentation of various dermatological conditions, making it imperative for healthcare professionals to be aware and knowledgeable of these differences to improve the accuracy of diagnoses and facilitate timely and appropriate treatment. One example is the presentation of necrotizing fasciitis in patients with darker skin tones, which can be distinct from that seen in individuals with lighter skin. Due to differences in skin tone, necrotizing fasciitis can present as a less noticeable discoloration in darker-skinned individuals, leading to potential diagnostic delays and poorer clinical outcomes.

To provide clinicians with a comprehensive portrayal of the clinical manifestations of infected aneurysms and necrotizing fasciitis, Table 1 can serve as a valuable reference. This table summarizes the findings described in the preceding sections, which may aid in the prompt and accurate diagnosis as well as the timely management of these potentially life-threatening conditions.

**Table 1:** Clinical Manifestations of Mycotic Femoral Aneurysms and Necrotizing Fasciitis

Clinical Manifestation	Mycotic Femoral Aneurysm	Necrotizing Fasciitis
Fever	+	+
Pain	+	+
Edema	+	+
Erythema	+	+
Warmth	+	+
Pulsatile mass	+	–
Bruit	+	–
Bullae	–	+
Skin necrosis	–	+
Ecchymosis	–	+
Subcutaneous crepitus	–	+
Rapidly progressing	–	+

Diagnostic Tools

The diagnosis of a mycotic aneurysm is based on a combination of clinical, imaging, and laboratory findings. While no specific algorithm exists for diagnosing an infected femoral aneurysm, imaging is the most reliable diagnostic tool, particularly a contrast computerized tomography. CT scans can reveal specific features such as lobulated contours, inflammation, or air around the blood vessel, and perianeurysmal fluid collection. Alternatively, contrast-enhanced MR angiography and digital subtraction angiography can be used, however, these methods are more invasive than a CT. In addition, elevated inflammatory laboratory results such as increased levels of C-reactive protein, leukocytes, erythrocyte sedimentation rate, and positive blood culture are suggestive of mycotic aneurysm. While histopathology can determine the presence of transmural inflammation which increases the likelihood of aneurysm rupture and spread of infection, it is not necessary for a diagnosis to be made. Cellulitis is an important differential diagnosis that should be considered if a mycotic aneurysm is expected [2].

Necrotizing fasciitis is a rare but life-threatening condition, with mortality rates ranging from 25-35%, which is increased in the presence of comorbidities such as immunosuppression, vascular disease, diabetes, alcoholism, and obesity [7]. Clinicians should maintain a high degree of suspicion for necrotizing fasciitis, and may use clinical scales like the laboratory risk indicator for necrotizing fasciitis (LRINEC) score to differentiate it from other soft tissue infections. A LRINEC score >6 is indicative of necrotizing fasciitis. The LRINEC is calculated based on the elevation of c-reactive protein, leukocytes, hemoglobin, sodium, creatine, and glucose. Although helpful in the diagnosis, the scale still requires a high index of suspicion, and imaging may be useful in the differential diagnosis. MRI or CT scans are the imaging modalities of choice for identifying the thickening of deep fascia, which is a hallmark of necrotizing fasciitis, however, thickening may not be present in the early stages of infection. Plain radiographs and ultrasound are considered a less effective modality in diagnosing necrotizing soft tissue infections but may be useful to rule out other possible modalities and visualize the presence of subcutaneous gas [17]. Exploratory surgery remains the most accurate method for diagnosing necrotizing fasciitis, and should not be delayed based on the availability of imaging or laboratory work [7]. 

Treatment

Treatment of a mycotic aneurysm can include surgical intervention followed by antibiotic treatment. Surgical intervention is the repair or the ligation of the mycotic artery with debridement following. If conditions require, amputation may be necessary. The biggest point of contention among surgeons is if revascularization should be done immediately or at a follow-up date [6]. In situ or immediate revascularization comes with an increased risk of infection of the newly grafted vasculature [18]. Most surgical compilations related to in situ repair come from the length of surgical time and ischemia distal to the operation site. Some such complications are cerebrovascular accidents, myocardial infarction, or kidney damage. Late complications of an in-situ repair can include reinfection and leakage caused by fistula development or anastomotic failure, which may lead to further complications. Some studies have reported poorer outcomes and high mortality rates associated with in situ repair, especially when the synthetic graft is not removed. In the case of ligation without immediate revascularization, the patient has an increased risk of debilitating claudication and risk of limb loss. After the surgical procedure, it is recommended to undertake a six- to eight-week regimen of antibiotic treatment, as outlined in Table 2. A combination of vancomycin with ceftriaxone, fluoroquinolone, or piperacillin-tazobactam to protect against gram-negative organisms is recommended [2]. The recommended surgical and antibiotic treatments for mycotic aneurysms are recommended in Table 2.

**Table 2:** Recommended Surgical and Antibiotic Treatments for Mycotic Aneurysms

Treatment of Mycotic Aneurysms	
Surgical Interventions	Antibiotic Treatment
Debridement with or without immediate revascularization	Vancomycin in combination with Gram-negative antibiotics
Revascularization options:	Gram-negative antibiotic options:
In-situ graft	Ceftriaxone (Rocephin)
Extra-anatomic graft	Piperacillin-Tazobactam (Zosyn)

The treatment of necrotizing fasciitis must be prompt and comprehensive. Antibiotic treatment alone, without surgical intervention, is associated with a high mortality rate. Therefore, exploratory surgery should be initiated immediately if necrotizing fasciitis is suspected. During surgery, samples should be obtained and cultured, and the infected area should be thoroughly debrided. If the infection is severe or uncontrolled, amputation may be necessary. Antibiotic therapy should be initiated immediately after surgery, even before culture results are available. Once culture results and sensitivity trials have been done antibiotic treatment should be tailored to those results. There are three types of necrotizing fasciitis based on the type of bacterial infection, with type 1 being polymicrobial and type 2 being monomicrobial. There is a lack of consensus on the subtypes for the diagnosis of necrotizing fasciitis. For the sake of this paper, we will consider three types; types 2 and 3 are both monomicrobial infections, type 2 is caused by a streptococcus infection, and type 3 is necrotizing fasciitis being etiologically linked to gram-negative bacteria, including but not limited to Vibrio vulnificus, Aeromonas hydrophila, and clostridium [12]. Recommended antibiotic treatment for different types of necrotizing fasciitis can be noted in Table 3. The Infectious Disease Society of America recommends the use of a combination of antibiotics for treatment. Type one polymicrobial infection should be treated with clindamycin in combination with a broad spectrum such as piperacillin-tazobactam or ceftriaxone-metronidazole. Type 2 necrotizing fasciitis is primarily caused by Group A streptococcus (GAS) bacteria which should be treated with clindamycin combined with MRSA coverage antibiotics such as Linezolid or Vancomycin. Linezolid may be a preferred choice in patients susceptible to kidney damage due to its lesser nephrotoxic effects than vancomycin [19]. Type 3 clostridium-infected necrotizing fasciitis should be treated with clindamycin combined with a broad-spectrum beta-lactam such as piperacillin-tazobactam or meropenem. The recommended antibiotic treatment for different types of necrotizing fasciitis is depicted in Table 3.

**Table 3:** Recommended Antibiotic Treatment for Different Types of Necrotizing Fasciitis MRSA: methicillin-resistant Staphylococcus aureus

Treatment of Necrotizing Fasciitis Antibiotic Treatment- Based on Infection Type		
Type 1 Polymicrobial	Type 2 Streptococcus	Type 3 Clostridium
Clindamycin + Broad-spectrum	Clindamycin (900mg IV Q8Hr) + MRSA Coverage	Clindamycin (900mg IV Q8Hr) + Broad-spectrum beta lactam
Broad-spectrum:	MRSA Coverage Options:	Broad-spectrum beta lactam options:
Piperacillin- tazobactam	Linezolid (600m IV q12Hr) *preferred	Piperacillin-tazobactam *preferred
Ceftriaxone–metronidazole	Vancomycin	Meropenem

## Review

Discussion

Over the years, there has been an evolution in the methods of identification, interventions, and treatment options for necrotizing fasciitis secondary to mycotic femoral aneurysms, resulting in improved outcomes for affected individuals. The identification of risk factors in combination with clinical features is important in the evaluation of both mycotic aneurysms and necrotizing fasciitis. The diagnosis of mycotic aneurysms is not straightforward and may require a combination of clinical, laboratory, imaging, and intraoperative findings [20]. Imaging modalities that are commonly used for the diagnosis of aneurysms include contrast-enhanced CT scan and contrast-enhanced MR angiography. Although digital subtraction angiography can provide similar information, it is a more invasive imaging modality [21].

Clinical assessment remains as the primary method for diagnosing necrotizing fasciitis. Necrotizing fasciitis may initially present with malaise, diarrhea, pain and fever, which has been misdiagnosed as muscle cramping, deep vein thrombosis and food poisoning [12]. Imaging can be useful when the diagnosis is uncertain. Plain film imaging can show increased soft tissue thickness and opacity resembling cellulitis, but CT scans are more sensitive in identifying these infections. CTs will expose gas in the tissue if the infection is caused by necrotizing fasciitis. Type 1 but should be differentiated from gangrene. X-rays are generally not useful in making the diagnosis of necrotizing fasciitis. In some cases, local anesthesia may be used to probe the affected area for signs of necrotizing tissue, which often offers little resistance to penetration. Aspiration and gram staining can also be performed [1]. Recent studies have also shown that point-of-care ultrasound can be used for the diagnosis of necrotizing fasciitis, with good specificity and sensitivity [22]. Despite this, further research is needed to establish the utility of this modality in the diagnosis of this condition. Clinical assessment will be needed to differentiate between necrotizing fasciitis and non-necrotizing cellulitis which is quite common in diabetic patients. Patients with necrotizing fasciitis will have systemic manifestation of sepsis, such as tachycardia, acidosis, hyperglycemia, and leukocytosis. Laboratory risk indicator for necrotizing fasciitis (LRINEC) is a useful tool in diagnosing and differentiating necrotizing fasciitis, a score >6 is indicative of necrotizing fasciitis. The LRINEC test had a positive predictive value between 57 and 92% across three studies. However, caution should be used when assessing pediatric populations where the average LRINEC score is 3.7, below the diagnostic threshold [12].

To effectively manage mycotic aneurysms, treatment with antibiotics plays a pivotal role, and is tailored to address the specific causative organism [23]. Over time, because of the advancements in antibiotic use, antibiotic regimens for treating necrotizing fasciitis secondary to mycotic aneurysms have been updated, with broad-spectrum antibiotics taking the forefront empirically, until culture results and sensitivities have been obtained. These broad-spectrum antibiotics as mentioned before include clindamycin, piperacillin-tazobactam, vancomycin, ceftriaxone-metronidazole, and meropenem [3]. Previously, clindamycin was used as a monotherapy for necrotizing soft tissue infections but because of its suboptimal concentrations as well as increasing resistance incidence, particularly in the United States, it has been advised to be a part of combined therapy, with the medication mentioned above [24]. To our dismay, as seen with clindamycin, as time progresses there are also more and more vancomycin antibiotic resistance cases in addition to adverse effects, as such it has been continuously replaced with the use of linezolid [19].

Additionally, regarding antibiotic therapy, some cases have been identified to require prolonged exposure after the initial six to eight-week IV (intravascular) administration, such as those who utilized prosthetic grafts for arterial reconstruction [3]. Unfortunately, the advantages of these long-term antibiotic use following surgical intervention have not been studied sufficiently to be confirmed. However, the same study did compare the rates of recurrent infection, morbidity, and mortality between the use of regular prosthetic grafts and antibiotic-soaked Dacron grafts during arterial reconstruction, with the Dacron graft showing a decrease in these rates from 25% to 16-20% overall. Furthermore, autologous grafts, being donated by the recipients themselves have also been shown to have low reintervention rates as well as high resistance to infection rates [3].

Surgical procedures are also needed to remove all affected tissue following a mycotic aneurysm. It has been described as a two-part process with arterial ligation and debridement being the first followed by reconstruction or revascularization of the affected area. Two surgical options are available for reconstruction: extra-anatomic or in-situ reconstruction [25]. Extra-anatomic reconstruction involves multiple operations to restore normal blood flow, while in-situ reconstruction provides a definitive solution in a single operation [26]. However, in-situ repair has been avoided due to concerns about graft or anastomotic destruction by prolonged infection [27]. In recent years, endovascular repair has emerged as a viable alternative to open surgery, especially for individuals with high surgical risks [28]. While revascularization processes remain necessary in treating necrotizing fasciitis secondary to mycotic aneurysms, over time, it has been identified that initial surgical intervention should solely focus on ligation and debridement only, with the revascularization process as a follow-up [2]. This order of treatment has increased the incidence of limb loss in these patients to 3.8% but weighing it with a decreased rate of morbidity and mortality allows for justification. It is also important to note that success rates of revascularization in comorbid patients such as diabetics or intravenous drug users have decreased in comparison to patients without such underlying diseases [6]. 

Necrotizing fasciitis requires prompt management for optimal outcomes. Early surgical debridement of all affected tissues is essential, and reconstruction of the wound including vasculature can only be considered after the infection is resolved [29]. Negative pressure therapy has been shown to be useful in improving tissue oxygenation, reducing bacterial count, and promoting granulation tissue formation [30]. This therapy is particularly useful for constant debridement and wound cleanliness in cases where conventional techniques have failed [31]. The use of a vacuum-assisted closure (VAC) system has also been demonstrated for temporizing wounds, allowing for less complex reconstructions of major soft tissue defects [32]. Hyperbaric oxygen therapy is currently under investigation for its potential role in the treatment of necrotizing fasciitis. However, it is not widely available and would be ineffective for type 2 necrotizing fasciitis caused by streptococcus, which is an aerobic bacteria [19]. Another current treatment option being explored is the use of intravenous immunoglobulin therapy to neutralize toxins and reduce the further development of infection. In one study with 4,127 patients with necrotizing fasciitis across 130 hospitals, IVIG had no effect on patient mortality or length of stay. Further studies would need to be conducted in the future to fully evaluate the benefits of IV immunoglobulin therapy [12].

## Conclusions

This paper sought to summarize a literature review conducted on necrotizing fasciitis secondary to mycotic femoral aneurysms. It focused on any changes throughout the years with respect to pathophysiology, clinical features, investigations as well as interventions and management. The exact pathophysiology of the disease remains unclear but studies continue to attribute it to the cellular and molecules processes, which starts with bacterial seeding and ends with the release of enzymes, endotoxins and exotoxins ultimately being responsible for the spread of infection to surrounding tissues inclusive of both superficial and deep fascia. Investigations have remained largely unchanged with CT/MRI scans and cultures to identify soft tissue infections, while angiography can also be used to identify the site of arterial damage. However, surgical exploration is still the definitive test in diagnosing the presence of necrotizing fasciitis secondary to mycotic femoral aneurysms.

The central component surrounding the management of these patients have not changed with antibiotic therapy and surgical interventions being the primary treatment modalities. However, many studies have identified decreasing morbidity and mortality by changing the orders of these interventions as time progresses. In conclusion, not many changes were observed over the years regarding the overall disease of necrotizing fasciitis secondary to mycotic femoral aneurysms. Nonetheless, an updated literature review in the future will be beneficial to those interested in this topic.
